# Bioenergetic Measurements in Children with Bipolar Disorder: A Pilot ^31^P Magnetic Resonance Spectroscopy Study

**DOI:** 10.1371/journal.pone.0054536

**Published:** 2013-01-30

**Authors:** Elif M. Sikoglu, J. Eric Jensen, Gordana Vitaliano, Ana A. Liso Navarro, Perry F. Renshaw, Jean A. Frazier, Constance M. Moore

**Affiliations:** 1 Department of Psychiatry, University of Massachusetts Medical School, Worcester, Massachusetts, United States of America; 2 Department of Psychiatry, McLean Hospital/Harvard Medical School, Belmont, Massachusetts, United States of America; 3 Deparment of Psychiatry, University of Utah, Salt Lake City, Utah, United States of America; RIKEN Brain Science Institution, Japan

## Abstract

**Background:**

Research exploring Bipolar Disorder (BD) phenotypes and mitochondrial dysfunction, particularly in younger subjects, has been insufficient to date. Previous studies have found abnormal cerebral pH levels in adults with BD, which may be directly linked to abnormal mitochondrial activity. To date no such studies have been reported in children with BD.

**Methods:**

Phosphorus Magnetic Resonance Spectroscopy (^31^P MRS) was used to determine pH, phopshocreatine (PCr) and inorganic phosphate (Pi) levels in 8 subjects with BD and 8 healthy comparison subjects (HCS) ages 11 to 20 years old.

**Results:**

There was no significant difference in pH between the patients and HCS. However, frontal pH values for patients with BD increased with age, contrary to studies of HCS and the pH values in the frontal lobe correlated negatively with the YMRS values. Global Pi was significantly lower in subjects with BD compared with HCS. There were no significant differences in PCr between the groups. Global PCr-to-Pi ratio (PCr/Pi) was significantly higher in subjects with BD compared with HCS.

**Conclusions:**

The change in Pi levels for the patients with BD coupled with the no difference in PCr levels, suggest an altered mitochondrial phosphorylation. However, our findings require further investigation of the underlying mechanisms with the notion that a mitochondrial dysfunction may manifest itself differently in children than that in adults.

**Limitations:**

Further investigations with larger patient populations are necessary to draw further conclusions.

## Introduction

In recent years, it has been suggested that Bipolar Disorder (BD) may be a disorder of mitochondrial dysfunction [1], [2], [3]. In 2004, Konradi et al. reported abnormal regulation of nuclear genes coding for mitochondrial proteins in BD [4]. Such a deficit in coding for mitochondrial proteins would mainly affect the respiratory chain, causing energy metabolism dysfunction and cellular degeneration [5]. How a dysfunction in energy metabolism manifests itself clinically depends on many parameters and indeed different or even the same mitochondrial dysfunction may have different clinical manifestations [5].

Phosphorous Magnetic Resonance Spectroscopy (^31^P MRS) is a method that allows for the measurement of pH, high-energy phosphates, and phospholipid metabolism *in vivo*. Kato and colleagues, throughout the 1990s and early 2000s, published a number of studies showing altered pH (measured using ^31^P MRS) in adults with BD. In particular, they showed reduced pH in euthymic patients with BD [6], [7], [8], [9], [10]. These studies, coupled with other findings that showed increased lactate (measured using proton (^1^H MRS) in BD [11], suggest reduced pH as a consequence of increased lactate levels due to a shift away from oxidative phosphorylation towards glycolysis [3]. This shift may be a consequence of mitochondrial dysfunction. Reductions in brain mitochondrial function may be manifested by decreased phosphocreatine (PCr) and increased inorganic phosphate (Pi), measured using ^31^P MRS [12]. Through their work on skeletal muscle energetics, Chance et al. have suggested that the PCr-to-Pi ratio (PCr/Pi) may be a sensitive marker of mitochondrial dysfunction [13].

To the best of our knowledge, there are no published studies using ^31^P MRS to investigate pH in children and adolescents with BD. We are unaware of any studies that have reported the PCr-to-Pi ratio (or Pi) in BD. The first aim of this study was to replicate the Kato studies in children and adolescents with BD to investigate pH levels and putative mitochondrial dysfunction. Kato et al. noted that while pH was lower in euthymic patients with BD, patients who were manic or depressed had higher pH levels compared with healthy comparison subjects (HCS) or euthymic patients [7], [8]. Based on these studies, we hypothesized that pH would be higher in manic, mixed, or depressed children and adolescents with BD compared with HCS or euthymic subjects with BD. In addition, higher pH levels would be associated with levels of mania (measured using the YMRS) and depression (measured using the CDRS) in the children with BD. The second aim of this study was to investigate the PCr/Pi in BD. We hypothesized that, consistent with a mitochondrial dysfunction, PCr would be reduced, Pi increased, and PCr/Pi reduced in children and adolescents with BD, compared with HCS.

## Materials and Methods

### Subjects

The Institutional Review Boards at both McLean Hospital and the Cambridge Health Alliance approved this study. All subjects were recruited through McLean Hospital (outpatient, partial and inpatient programs) and online advertising. All of the children (with one exception) were interviewed with the Schedule for Affective Disorders and Schizophrenia for School-Age Children (KSADS-PL) by a child psychiatrist. For the purpose of this study, we differentiated between the episodes and the current mood state at a given time point. The past episodes and the diagnoses of BD in the children, adolescents and young adults, based on unmodified Diagnostic and Statistical Manual of Mental Disorders (DSM-IV TR) criteria, were made following the clinical interviews. One patient was an inpatient at McLean Hospital at the time of the study, this subject was not interviewed with the KSADS-PL due to scheduling issues. This subject had a previous diagnosis of BD made by a child psychiatrist. The current mood state was determined in all of the subjects on the day of the imaging using the Young Mania Rating Scale (YMRS: [14]) and the Children’s Depression Rating Scale-Revised (CDRS: [15], [16]). (Manic: YMRS = 12 or above; CDRS = 29 or below. Depressed: CDRS = 30 or above; YMRS = 11 or below. Mixed: YMRS = 12 or above; CDRS = 30 or above. Euthymic: YMRS = 11 or below; CDRS = 29 or below). Exclusion criteria included: a history of a uncontrolled general medical disorder; a history of neurological illness (including head trauma with loss of consciousness, seizure disorder, multiple sclerosis, cerebral ischemia or infarction, neoplasia); autism; schizophrenia; alcohol or drug dependence (during two months prior to scan or total past history of ≥12 months); mental retardation (full scale I.Q. <70 (historical measure)); electroconvulsive therapy; a contraindication to MR scan including metal fragments or implants; claustrophobia; lactation; pregnancy (all females of child bearing age passed a negative pregnancy urine test prior to scanning). Unique exclusion criteria for the healthy comparison subjects included an Axis I diagnosis and a family history of a mood disorder in a first degree relative. Family history of DSM-IV psychiatric diagnoses was obtained during the phone screen and during the clinical assessment and interview about the child from the parents.

After the study was described, all parents signed a written informed consent form and all children signed a written informed assent form. Adult study participants (18 years or older) signed a written informed consent form.

### MR Imaging and Spectroscopy

**Figure 1:**
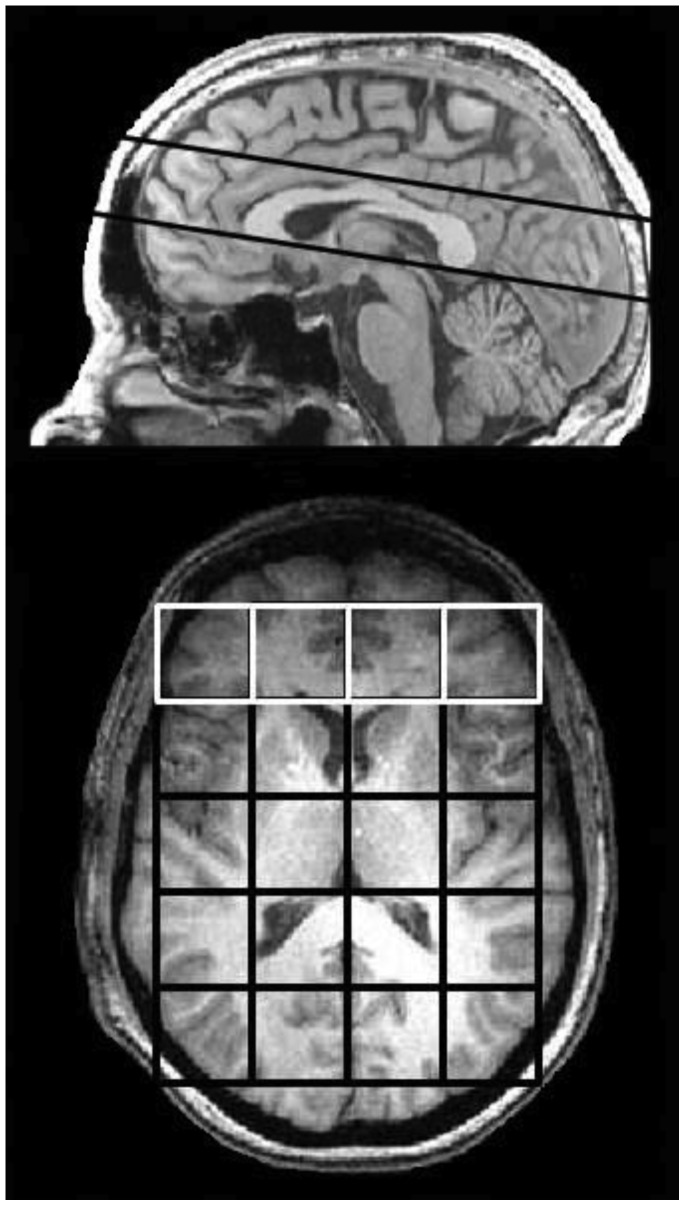
Slice location (top) where global chemical shift imaging grid (5×4 grid) and frontal lobe grid (1×4 grid) were located.

Magnetic Resonance Spectroscopy (MRS) studies were performed on a 4.0 T Varian Unity/Inova whole body MR scanner (Varian NMR Instruments, Palo Alto, CA) equipped with a dual-tuned proton-phosphorus head coil (Bioengineering Inc., Minneapolis, MN). Sagittal T1-weighted, magnetization-prepared 3D-FLASH (fast, low-angle shot) images were collected for chemical shift imaging (CSI) slab placement. An optimized phosphorus 2D-CSI, pulse-acquire sequence acquired ^31^P-CSI data from the mid-sagittally-placed slab (matrix size = 8×8, TR = 3 s; tip-angle = 80°; slice thickness = 3 cm; Rx bandwidth = ±2 kHz; complex-points = 1024; readout duration = 256 ms; NEX = k-space weighted; pre-acquisition delay = 1.2 ms; field of view (FOV) = 24 cm×24 cm; nominal voxel volume = 27 ml, scan-time = 23 min). Effective voxel-volume for ^31^P-CSI was defined as the cylindrical volume bound by the FWHM of the point-spread function (PSF) and estimated to be ∼50 cc. For each scan, a 5-by-4 voxel grid was centered within the brain according to axial images that fell within the 3 cm thick slab. For the frontal analysis, the grid was positioned such that 4 voxels encompassed the entire frontal cortical region, just anterior to the genu of the corpus callosum. The four frontal voxels were in-line from left to right (see Figure 1).

### Data Processing and Analysis

**Figure 2:**
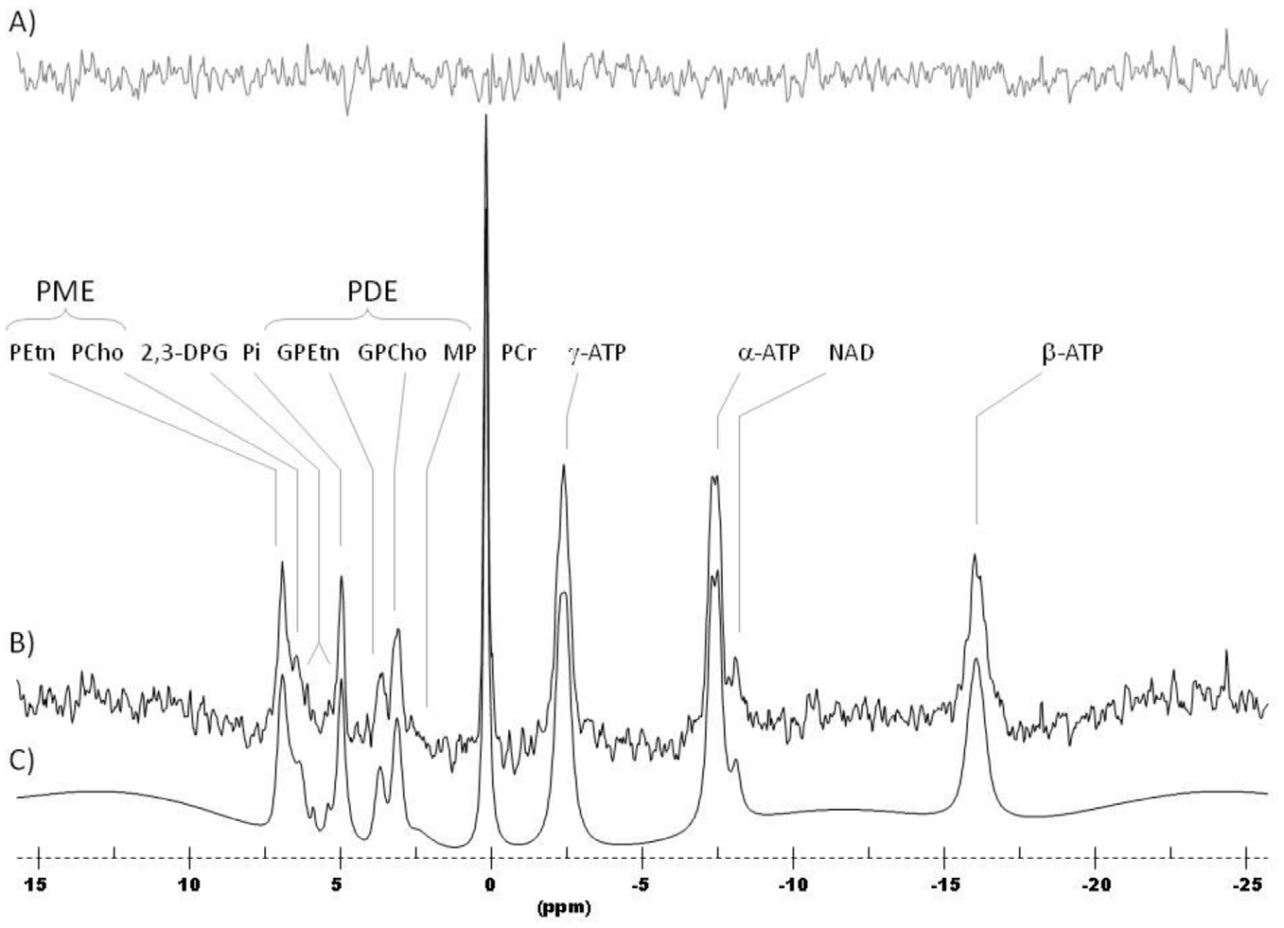
^31^P MR spectrum from a whole (global) brain slice. A) Residual of fit; B) ^31^P MR spectrum; C) Spectral Fit.

The ^31^P-CSI data were first read into a zero-padded 8×8 matrix (including the 5×4 sub-matrix containing the brain) and corrected by a set of scalar correction factors that correct each k-space sample for the discrepancy of defining an optimal, theoretical k-space filter with an integer number of averages for each phase-encode step. The corrected ^31^P-CSI data were then Fourier-Transformed to spatially-resolve each voxel throughout the brain. Each spatially-resolved spectrum was stored as a time-domain free-induction decay (FID) for each voxel (see Figure 2).

### Spectral Fitting

All ^31^P-CSI spectra were fitted individually on a voxel-by-voxel basis with a non-linear, iterative routine developed on-site, based on the Marquardt-Levenberg algorithm for non-linear, least-squares fitting of complex waveforms. The routine incorporates the use of prior spectral knowledge such as J-coupling constants, chemical-shifts and linewidths and applies pre-optimized constraints to converge on an optimal fit. The template that we used for ^31^P fitting at 4T models each metabolite as a series of Lorentzian and Gaussian lineshapes. In our template we have models that include phospocreatine (PCr), nucleoside triphosphate (NTP), and inorganic phosphate (Pi) as Lorenztians and phosphoethanolamine (PEth), phosphocholine (PCh), Phosphoserine (PSer), gylcerophosphoethanolamine (GPEth), phosphocholine (GPCh) and MP (membrane phospholipid) as Gaussians. For each metabolite, the raw peak areas returned by the routine were expressed as a ratio to total phosphorus, referring to the total integrated area under the entire modeled spectrum. Whole-slab (5-by-4 voxel grid) and frontal lobe ^31^P brain metabolite ratios normalized to total ^31^P signal per voxel were then derived from the ^31^P data. Whole brain and frontal lobe pH levels were determined by finding the chemical-shift difference between the inorganic-phosphate (Pi) and phosphocreatine (PCr) resonances. In addition, the metabolite values were averaged across all voxels to obtain the global measurements and across the frontal lobe voxels to obtain the frontal lobe measurements.

### Statistics

Statistical analysis was performed using SPSS 20 software package. Due to the small sample size non-parametric tests were used: the Mann-Whitney U Test to compare between groups differences, and Spearman’s rho for examining correlations. A p-value of 0.05 was considered significant.

## Results

Sixteen subjects participated in this study: 8 subjects who meet criteria for BD (age: 16.94±1.99 years; 2 female) and 8 HCS (age 15.53±3.42; 4 female). The subjects with BD had a mean YMRS of 11.56±9.02 and CDRS of 27.38±6.21, compared with 0.55±0.93 and 17.73±0.90, for the HCS subjects, respectively. Subject demographics are shown in Table 1. There was no significant difference in age between the groups. Three of the subjects with BD were euthymic, the remaining 5 were either mixed, manic or depressed. The YMRS and CDRS were significantly different between the groups: Mann-Whitney U test p<0.001.

**Table 1:** Demographic information for subjects who participated in this study.

Subject	Sex	Age(at scan)	Height(cm)	Weight(kg)	Race	Diagnosis	ComorbidDiagnosis	Lithium(Li)	Li Rx(mg)	OtherMedications	YMRS	CDRS
HCS 1	M	11.4	160	40.8	Caucasian	HCS	none	–	–	–	3	17
HCS 2	M	12.0	162.6	39.9	Caucasian	HCS	none	–	–	–	0	17
HCS 3	M	12.3	137.2	37.2	Caucasian	HCS	none	–	–	–	1	18
HCS 4	F	15.7	152.4	46.3	African-American	HCS	ADHD	–	–	–	0	17
HCS 5	F	16.0	154.9	51.7	Caucasian	HCS	none	–	–	–	0	17
HCS 6	F	16.8	167.6	61.2	Caucasian	HCS	none	–	–	–	0	18
HCS 7	M	19.8	190.5	79.3	Caucasian	HCS	none	–	–	–	0	19
HCS 8	F	20.2	160.2	59	African-American	HCS	none	–	–	–	1	19
BD 1	M	14.5	152.4	38.6	African-American	BD	N/A	Yes	N/A	3,4	24	37
BD 2	M	14.7	170.2	63.5	Caucasian	BD	OCD	Yes	900	1,2,9	17	25
BD 3	M	15.7	180.3	82.6	Caucasian	BD	ADHD,PTSD	Yes	1000	10,11,12,6,13	4	19
BD 4	M	17.0	172.7	62.6	Caucasian	BD	ADHD,OCD,ODD	Yes	520	5,6,2,4	6	31
**BD 5	M	17.3	177.8	63.5	African-American	BD	ADHD	Yes	450	7,8	1	20
BD 6	F	20.4	162.6	58.1	Caucasian	BD	PTSD,Bulimia	Yes	900	5	5	26
**BD 7	M	17.2	175.3	84.8	African-American	BD	N/A	No	0	8	12	33
**BD 8	F	18.7	167.6	70.3	Caucasian	BD	none	No	0	15,2	23.5	28

(KEY: **Other Medications:** 1 = risperidone, 2 = lamotrogine, 3 = atomoxetine, 4 = quetipine, 5 = valproate, 6 = dexmethylphenidate, 7 = clonodine, 8 = methylphenidate, 9 = bupropion, 10 = benzatropine, 11 = paliperidone, 12 = desipramine, 13 = clonazepam, 14 = fluvoxamine, 15 = citalopram; ** = drug test positive for THC).

HCS: Healthy comparison subject. BD: Subjects with Bipolar Disorder. ADHD: Attention Deficit Hyperactivity Disorder. ODD: Oppositional Defiant Disorder. OCD: Obsessive–Compulsive Disorder. PTSD: Post-Traumatic Stress Disorder. For other abbreviations see text.

### pH

**Figure 3:**
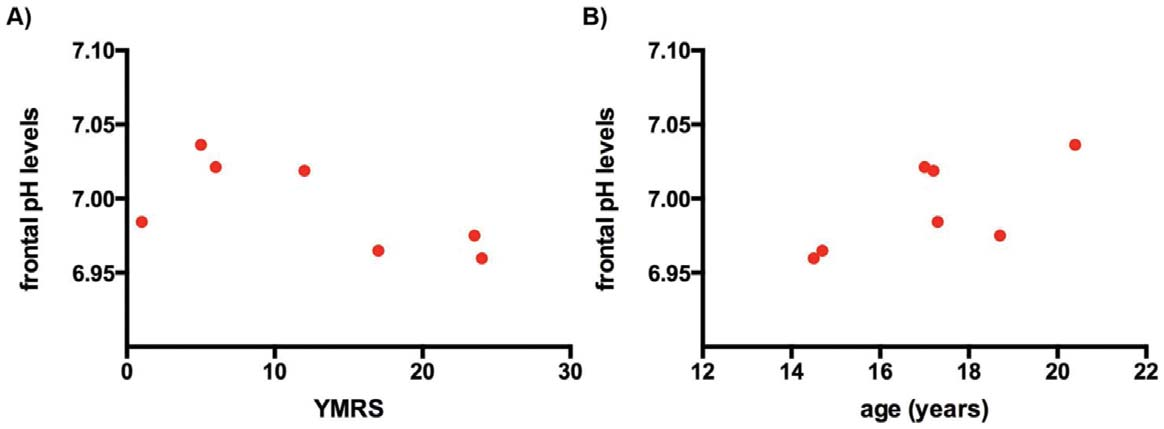
Scatter plot of frontal pH levels A) against the Young Mania Rating Scale (YMRS), B) against the ages for the Bipolar Disorder patients (n = 7).

pH was not significantly different between the two groups. Global pH was 7.00±0.02 in subjects with BD (N = 8); and 7.00±0.02 in HCS (N = 8). Frontal lobe pH values were 6.99±0.03 in subjects with BD (N = 7) and 6.99±0.03 in HCS (N = 7). There were no significant difference in pH between the euthymic children with BD and the depressed/manic/mixed mood children; or between the HCS and the depressed/manic/mixed mood children. In the subjects with BD the YMRS correlated negatively with pH in the frontal lobes (Spearman’s rho = −0.75, p = 0.05, N = 7) (see Figure 3A). There was no correlation between the CDRS and pH in either region. In addition, there was a positive correlation between the age and the frontal pH levels only for patients (Spearman’s rho = 0.64, p = 0.12, N = 7) (see Figure 3B).

### PCr-to-Pi Ratio (PCr/Pi)

The global PCr/Pi was significantly higher in subjects with BD compared with HCS (Mann-Whitney U-test, p = 0.05). There was a trend for frontal lobe PCr/Pi to be higher in subjects with BD compared with HCS (p = 0.18). Global PCr/Pi was 2.84±0.69 in subjects with BD (N = 8); and 2.35±0.31 in HCS (N = 8). Frontal lobe PCr/Pi values were 2.89±0.91 in subjects with BD (N = 7) and 2.40±0.54 in HCS (N = 7).

There were no significant difference in PCr/Pi between the euythmic children with BD and the depressed/manic/mixed mood children. However the depressed/manic/mixed mood children retained the higher global PCr/Pi compared with HCS (p = 0.08).

For the subjects with BD there was no correlation between YMRS and CDRS and either region’s PCr/Pi.

### Pi

Global Pi was significantly lower in patients with BD compared with HCS: 0.066±0.005 in BD (N = 8) and 0.076±0.009 in HCS (N = 8) (Mann-Whitney U-test, p = 0.021). In addition, global Pi was significantly lower in depressed/manic/mixed mood children (0.065±0.006, N = 5, p = 0.028) compared with HCS. Analysis of frontal lobe Pi values showed no significant difference between any of the experimental groups.

### PCr

There was no significant difference in global or frontal lobe PCr values between either of the experimental groups.

## Discussion

Contrary to our hypothesis, there was no significant difference in pH between the two groups. In a ^31^P MRS study, Rae et al. demonstrated that pH decreases in the frontal lobes of healthy subjects with age (98 males, ages 6 to 72 years old) [17]. However, our findings show that even though we only have data from 8 patients, the pH ranges from 6.96 to 7.04 in the frontal lobes of the BD patients, which is an increase of 1.14%. Therefore, despite there being no significant difference in pH between the groups, pH values in BD patients increased with age rather than the expected decrease. This is perhaps related to the fact that the younger subjects with BD in the study having the higher YMRS values: because contrary to our hypothesis lower pH was associated with higher YMRS values.

These ^31^P MRS results are in agreement with our previous ^1^H MRS study demonstrating reduced glutamine (Gln) in the anterior cingulate cortex (ACC) of non-medicated children with BD (all of whom were mixed: manic and depressed) compared with HCS [18]. The brain metabolism of glutamate and glutamine is pH sensitive. Alkalinization of astrocytes results in increased uptake of glutamate and glutamine synthesis [19]. This result is consistent with the result here - higher YMRS values being associated with lower pH and lower pH associated with lower glutamine synthesis.

We did not observe any significant differences in PCr, however global Pi was lower for patients with BD, and PCr/Pi was higher for the patients compared with the healthy subjects. This finding was contrary to our second hypothesis and the expected result of reduced PCr accompanied by an increase in Pi levels.

A possible explanation for the lower Pi values for the BD patients may be the fact that 6 out of the 8 patients were on lithium treatment, while 3 of the patients with BD tested positive for Tetrahyrdocannabinal (THC). Lithium has been also shown to reduce Pi in healthy subjects treated with lithium over a 14-day period (measured using ^31^P MRS [20]). In addition, THC has been shown to increase glial Ca^2+^ [21], which may lead to reduced Pi levels [19]. Therefore the results seen here for Pi may be a consequence of lithium and/or THC intake.

Nevertheless, the change in Pi levels coupled with the no difference in PCr levels, as observed in the patients with BD, suggests an altered mitochondrial phosphorylation [22], [23].

As stated in the introduction, how a dysfunction in energy metabolism manifests itself clinically depends on many parameters and indeed different, or even the same mitochondrial dysfunction may have different clinical manifestations [5].

### Conclusions

In this study, we aimed to investigate the patients with BD using ^31^P MRS, to understand the possible underlying mitochondrial dysfunctions for children and adolescents with BD.

Our results showed no significant difference in pH levels between the patients and HCS. However, pH levels acquired from the frontal lobes of BD patients increased with age, contrary to studies in HCS. Moreover, in the subjects with BD the YMRS correlated negatively with pH in the frontal lobes. Global Pi was significantly lower in patients with BD compared with HCS. Since we did not observe any significant difference in PCr between the groups, global PCr/Pi was significantly higher in patients with BD compared with HCS. The change in Pi levels for the patients with BD coupled with the no difference in PCr levels, suggest an altered mitochondrial phosphorylation.

### Limitations and Future Directions

The inclusion of only 16 subjects gave the study a limited capability to represent a realistic population of healthy subjects and subjects with Bipolar Disorder. In addition, even though all patients had a confirmed diagnosis of Bipolar Disorder, the patient group consisted of depressed/manic/mixed mood children as well as three euthymic children. One subject did not have a KSADS-PL interview at the time of the study (but had a diagnosis of BD) and they were unwilling to return for the evaluation. An analysis conducted excluding this subject did not alter the significant results of the study. In addition, all but two subjects were medicated with lithium, and three subjects tested positive for THC including the one non-medicated subjects with BD; this may have a confounding effect on the results. Given the small sample size we were unable to conduct a data analysis excluding these subjects. Finally, tissue differences, i.e. white vs. gray matter vs. CSF content, may possibly be a confounding variable, which needs to be further studied using a high resolution anatomical images. However, due to the global nature of the analysis, we would not expect to see any difference between the patients and healthy comparison subjects.
